# Developing welfare parameters for African elephants (*Loxodonta africana*) in fenced reserves in South Africa

**DOI:** 10.1371/journal.pone.0264931

**Published:** 2022-03-24

**Authors:** Marion E. Garai, Tenisha Roos, Tamara Eggeling, André Ganswindt, Yolanda Pretorius, Michelle Henley

**Affiliations:** 1 Elephant Reintegration Trust, Port Alfred, South Africa; 2 Mammal Research Institute, University of Pretoria, Pretoria, South Africa; 3 Southern African Wildlife College, Hoedspruit, South Africa; 4 Applied Behavioural Ecology and Environmental Research Unit, University of South Africa, Pretoria, South Africa; 5 Elephants Alive, Hoedspruit, Limpopo, South Africa; Institute of Animal Science, CZECH REPUBLIC

## Abstract

South Africa has many fenced reserves harbouring small to medium sized populations of African elephant (*Loxodonta africana*), most of which have been translocated. Elephants on fenced reserves may be exposed to various management interventions and practices (translocation, hunting, darting, high tourism impact, contraception programs, disruption due to infrastructure maintenance, etc.). These factors may impact the welfare of elephants. Poor elephant welfare may have serious consequences such as increased inter- and intra-species aggression that could result in fatalities. This is the first study to attempt to define behavioural and physiological welfare parameters for free-ranging elephants on small to medium sized reserves. The eight study sites incorporated reserves with different social structure combinations, elephant life-histories, reserve sizes, habitat, management, and tourism intensity. Data collection consisted of behavioural observations (10-minute videos) as well as faecal samples. By incorporating both behavioural and physiological (faecal glucocorticoid metabolite (fGCM) concentration) parameters, we aimed to investigate whether the two parameters showed similar trends. Five behavioural categories were identified (Arousal, Assessing, Ambivalent, Ambivalent/ Body care, and Frustrated behaviour), with various detailed behaviours demonstrated by the elephants that may indicate the influence of anthropogenic disturbance and possibly impact on animal welfare. The study showed significant differences between the selected detailed behaviours, behavioural categories and fGCM concentrations of elephants across the eight reserves. History seemed to be a decisive factor, as reserves with predominantly ex-captive elephants showed higher frequencies of certain behaviours as well as higher fGCM concentrations. Age, sex, reserve size and season were also found to contribute to our defined welfare indices and fGCM concentrations. This indicates that behavioural parameters, indicative of certain behavioural states, are valuable indicators of welfare, as supported by the physiological response of the elephants. The results also highlight the importance of taking multiple specified behaviours from a category into consideration when evaluating the welfare of elephants, to account for individual variation.

## Introduction

South Africa is in the unique position of having many small private and State wildlife reserves, which harbour various sized groups of elephants (*Loxodonta africana*) [1]. Apart from the large undisturbed elephant populations found within the Kruger National Park (KNP) and the adjacent reserves which have dropped their adjoining fences (collectively known as the Greater Kruger National Park (GKNP), as well as Tembe Elephant Park, and Addo Elephant Park, all others have introduced their elephants through translocation from either Kruger National Park or other private or smaller State reserves, with one reserve receiving some elephants from Zimbabwe. These translocated populations have been more or less successfully reproducing, some to the extent that they now have reached their desired ecological carrying capacity and managers are using several options to control the population numbers, such as range expansion, translocation and contraception [2].

The future of African elephants in general is looking bleak, with the savanna elephant (*Loxodonta africana)* classified as ‘Endangered’ and the forest elephant (*Loxodonta cyclotis*) as ‘Critically Endangered’ [3, 4], due to climate change, human encroachment [5], poaching [6], decreased available habitat [7], farming and trade [8], culling [9], and increased Human-Elephant-Conflict due to burgeoning human populations competing for the same resources [10, 11]. Moreover, in the South African context, influences which arise in small, fenced elephant populations and typical management interventions may also impact the welfare of elephants as these smaller to medium sized reserves often require a more intense management style. The social group type within these reserves post translocation could be incomplete (e.g. one age group missing) or fragmented (e.g. skewed sex distribution or unrelated elephants), which may result in particular welfare concerns. Furthermore, elephants within small to medium sized reserves in South Africa are often subjected to additional translocation events, darting, scientific research, hunting and lethal measures for problem animal control, high tourism impact, contraception programs, constant infrastructure maintenance and potentially perpetuated skewed demography. Therefore, we can expect these elephants to be negatively affected, as a result of a compromised environment, which could be reflected in changes to behavioural patterns.

Managers of smaller, fenced reserves frequently report elephants displaying aggression towards vehicles and people, breaking out of reserves or even a few fatal attacks on other elephants. This may indicate that their welfare is compromised, indicating that welfare considerations should be part of management practices on fenced reserves. Much literature has been published on the welfare of captive elephants [12–14], and models have been developed to assess captive elephant welfare such as the Five Domains Model [15] and a behavioural assessment tool [13, 16]. Yet to date, no such assessment tool has been developed for free-roaming elephants despite a clear need.

Globally there is growing interest in the necessity for understanding the welfare of wildlife [8, 17–20], and elephants in general are increasingly showing signs of stress or aggression, often resulting in increased Human-Elephant-Conflict. The growing awareness that welfare of free-ranging elephants is an important factor supports the One Welfare concept [20], and could improve the safety of both elephants and people in light of human interventions and reduced space. Considerable elephant research to date primarily focuses on the effects of elephants on the environment, however, it is crucial that we also consider the effects on the elephants of the man-made environment, as well as management and tourism effects. The terms ‘management and tourism’ *per se* imply intrusion on the elephant population especially in smaller reserves and could lead to serious consequences such as aggressive behaviour, which should not be ignored. Animals such as elephants with highly developed cognition, have extremely complex social systems [21]. The more complex and differentiated such a system is, the more sensitively it will respond to human interventions [22].

Welfare varies over a continuum ranging from poor to good, and can be addressed at behavioural [31], emotional [23], physiological [24], and neural levels [25]. A prolonged exposure or frequent perception of stressors has been postulated to indicate poor welfare, but authors differ on defining when the activation of the hypothalamic-pituitary-adrenal axis–the stress response–will entail negative consequences. Natural environments include fear and stress, so species have adapted by natural selection to cope with acute stressors [26]. It can be argued, therefore, that a certain amount of stress should be part of the lives of animals. However, chronic stress can have severe health consequences [27] and may influence behaviour patterns [28]. Moberg [29] suggested that stress becomes *distress* when it incurs a biological cost so large that the animal needs to divert biological resources (e.g. energy) away from normal biological functions, causing the adverse effects [29], such as failed reproduction, stunted growth, and increased disease susceptibility [27].

Welfare in animals is typically measured via physiological and behavioural responses [30]. However, such science-based approaches have predominantly been utilized for captive and domesticated animals (Table 1), where many assessment techniques have been developed [13, 16, 31–33]. Some possible indicators of welfare have been suggested in the captive elephant literature [13, 16], but these are not easily transferred o wild elephants and /or may be impractical for reserve managers to implement. As such, assessment of free-ranging elephant welfare to date has focussed on measuring stress-related hormones [34, 35], or discussing ethics [36]. Given the current lack of welfare parameters that can be applied to wild or free ranging elephants, management decisions run the risk of inadvertently harming individuals. Until there are precise welfare indicators, we remain uninformed as to what levels of anthropogenic stress or social disruption is acceptable to elephants.

**Table 1:** The collation of potential elephant welfare parameters developed to date and applicable to free-ranging elephants.

Type of behaviour	Description	References
**Arousal behaviour**	Indicator of nervousness, fear or anxiety (may or may not culminate into stress), but also positive arousal (social encounters and interactions, obtaining a desired goal etc): with typical behaviours in a gradient from low arousal (listening, ears spread, head held high, tail held up, walk around) to high arousal (running, cluster formation, aggression, vocalisation, often associated excessive temporal gland secretion, diarrhoea).	[37, 38]
Low arousal		
High arousal		
**Temporal Gland Secretion**	**(TGS)** is not necessarily an indication of a negative experience; shown in various arousal reactions. Immediate reaction to a cause. The dry streak on the cheek is visible for quite a while; if fresh and seen constantly one can assume the animal is in constant stress.	[37, 39]
	Social Stress: elephants at the top of the hierarchy (e.g. matriarchs) show increased TGS. This is not the same as the TGS in musth bulls.	
**Vocalisations**	Express emotions–positive and negative. Types of vocalisations and behavioural correlates indicate the valence (positive/negative) and arousal level. Especially calves in distress will emit loud vocalisations such as bellowing and screaming.	[40, 41]
**Decreased Responsiveness**	When all copying strategies fail, the elephant may go into depression or a state of apathy.	[29, 42, 43]
**Change of behaviour pattern**	Stress becomes distress when it incurs a biological cost so large that the animal needs to divert biological resources (e.g. energy) away from normal biological functions. There might be increased vigilance behaviour, or alternatively less diverse behaviour patterns and decreased responsiveness (depression, apathy).	[6, 44–47]
	Elephants change their activity patterns and range behaviour to become more nocturnal and increase their flight behaviour in areas where poaching occurs.	
	They travel faster and more directional outside of protected areas.	
**Abnormal behaviour**	Abnormal behaviour differs in pattern, frequency, or context from that which is shown by most members of the species in conditions that allow a full range of behaviour.	[47]
**Lack of range use**	Elephants with higher levels of glucocorticoids (GCs) or its metabolites may utilise less of the available range than elephants with similar range size and lower GC levels. This supports the spatial refuge hypothesis, and the authors suggest that chronic stress is associated with restricted space use. Translocated elephants displayed this behaviour and related higher GC levels for up to 6 years following a translocation event.	[36]
**Avoidance/preference behaviour**	Stimuli, events or other elephants that are disliked or induce fear, anxiety or stress are typically avoided (e.g. human disturbance). Stimuli events and elephants that are preferred will be sought out.	[48]
**Loss of variability and complexity**	One measure of behavioural stress is loss of variability and complexity of exploratory behaviour, as more energy is spent on increased metabolic rate. Not immediately visible, but careful data on detailed behavioural elements can show it in the analysis.	[49]
**Social competence/incompetence**	Social competence is the capacity to react in a species-specific way to social interactions and the ability of individuals to regulate the expression of their social behaviour to optimise their social relationships. Social competence involves capabilities to perceive and process social information, and to behave most appropriately in a given social context. The social environment encountered early in life can affect the expression of various social behaviours later in life in situations such as competition, forming dominance hierarchies, care for the young, and mating success.	[50–53]
	When the ability to interact socially with the mother and other group members in infancy is prevented, normal functioning later in life is disturbed.	
	Social deprivation, specifically early separation from mother, results in development of severe and uncontrolled aggressive behaviour, intense anxiety reactions, inability to develop social relationships, unnatural startle responses, and lack of recognition of social signals and can be expressed in stereotypic behaviour.	
**Genetically fixed behaviours not achievable**	Every species has its specific genetically fixed general requirements, such as feeding, social, health, body care, locomotion, resting. These requirements elicit species-specific behaviour patterns to achieve the goal. Behavioural responses can be due to endogenous (e.g. diurnal rhythm, hormones) or exogenous factors (e.g. visual, tactile or acoustic stimuli by conspecifics). If the goal cannot be reached by any species-specific behaviour, the animal can respond with frustration, anger, or contrary, depression and apathy (e.g. hardly any response to social contacts, no initiative to explore, standing doing nothing.). One well-known means of response used by captive animals is to resort to abnormal behaviours such as stereotypies.	[42, 44, 54]
**Life expectancy**	Life expectancy appears to be shorter in captive elephants, where none reached age >50, whereas life expectancy in an African elephant population not targeted by poaching is up to 60 years. Whereas the causes of death in captivity are manifold, it is interesting to note that in the Addo population confinement may well have had an adverse effect on the longevity of the elephants.	[12, 55–57]
**Reproductive success**	A study on African elephants revealed that social bonds, group composition and poaching risk significantly influenced a female elephant’s stress physiology. The results suggest that a disrupted social group creates a chronic stress condition for elephants, and this affects the reproductive success, as well as growth and immunity.	[24, 58]

In this study we only use the term stress to indicate physiological changes measured with a stress-related biomarker i.e. significantly higher faecal glucocorticoid metabolite (fGCM) concentrations, and aimed to find specific behaviours that may indicate stress or potential stress. Although non-invasive monitoring of fGCM concentrations has been in practice for some time [35], we do recognise that this approach has its intrinsic challenges and respective concentrations can vary between individuals, e.g. due to reproductive state of males (i.e. musth) and females [59]. Yet it is crucial that we develop welfare standards and specifically identify behavioural parameters which will enable us to assess welfare issues in free-roaming elephant populations. As it is not always possible to conduct a detailed physiological study on free-ranging elephants, emphasis must be placed on behaviours which have been shown to correlate with welfare [8]. These behaviours must be easily visible to reserve managers or anyone assessing the elephants, within a reasonable time period. In assessing the welfare status of a group or population we must expect that even under identical environmental conditions, different individuals within that group or population may have a different welfare status [19].

The aim of this study was to identify behavioural indicators of reduced welfare and their endocrine correlates across various groups of African elephants. More specifically, the objectives of the study were a) to determine which behaviours could indicate arousal, nervousness, or frustration, which could be utilised as indicators of potential welfare concerns, and to compare these across reserves and b) to quantify faecal glucocorticoid metabolite concentrations in these elephant groups as a biomarker for physiological stress.

## Methods & materials

### Study location

The study included eight game reserves situated in South Africa. The reference reserve for this study (reserve 2 listed in Table 2) has a size of approximately 208 800-ha and forms part of the GKNP. Three of the remaining reserves are situated in the Eastern Cape, one in the Western Cape, one in Limpopo, one in KwaZulu-Natal and one in the North West Province. The study site selection aimed at incorporating reserves with different combinations of management intensity, tourist density, size and bioregion.

**Table 2:** Summary of the reserve size, bioregion, characteristics of the African elephant (*Loxodonta africana)* population and the average daily tourist density (high- and low tourist season) of the eight reserves included in the study.

Reserve	Reserve Size (ha)	Bioregions [60]	Elephant Population size	Elephant social structure [21]	Elephant population history	Percentage of previously captive elephants	Average tourist density per day	
							Guided Drives	Self-Drives
**1**	Small (5988)	Albany thicket	25	IC	W	0	11–20	0
2*	Large (208 800)	Lowveld	+/-3607	C	W	0	30–50	30–50
**3**	Medium (9000)	Fynbos	13	IC	W & PC	31	11–20	1–10
**4**	Small (2 792)	Albany thicket	43	C	W	0	21–30	0
**5**	Medium (10 000)	Central Bushveld	12	IC	W & PC	83	1–10	0
**6**	Small (4500)	Between Lowveld and Indian Ocean coastal belt	29	IC	W & PC	4	1–10	0
**7**	Small (4355)	Upper Karoo	9	IC	W	0	1–10	0
**8**	Large (90 000)	Kalahari Bushveld	8	IC	PC	75	0	1–10

Reserve size: Small = 500 – 8000ha; Medium: >8000–30 000ha and Large: >30 000ha [61–63]

*As it is an open system this number fluctuates per year and season

(IC = Incomplete social structure; C = Complete social structure; W = Wild elephants; PC = Previously captive elephants).

The selected reserves are representative of the current South African elephant population dynamics on fenced game reserves. These populations can either be socially complete or incomplete, missing certain family tiers (detailed in Table 3), and consist of either wild and/or reintegrated elephants (detailed in Table 2). An elephant population was defined as complete when it comprised third-tier familial units (separated clusters of second-tier core groups (regularly associated mother-calf units)) and included at least one bull older than 35 years [21]. These socially disturbed elephant populations could arise from translocations because of population size management, legal killing of dangerous or damage causing elephants, or even due to reserves only housing females to avoid reproduction.

**Table 3:** Factors recorded at the beginning of each individual behavioural observation (focal sample).

Factor	Level	Description
**Elephant identification**	ID	Elephant identification (If available)
**History of the individual**	Previously captive	Elephants that have been housed in captive facilities and/or participated in human interactions
	Wild	Elephants that have not had any organized human interaction or confined housing
**Sex**	Male (M)	Sex of individual
	Female (F)	
**Age**	Juvenile (J)	Juvenile: <8 years
	Sub-adult (S)	Sub-adult: 8–20 years
	Adult (A)	Adult: >20 years
**Elephant social structure**	Complete / incomplete	An elephant population was defined as complete, when it comprised of third-tier familial units (separated clusters of second-tier core groups (regularly associated mother-calf units) and included at least one bull older than 35 years [21]
**Season**	Wet season /	High rainfall season
	Dry season	Low rainfall season
**Distance from elephant**	<30m; >30m; >50m	Distance of elephant in the focal observation from the research vehicle
**Group spacing**	Bundled	Bundled together less than an elephant length away
	Close	Nearest neighbour on average between 5-20m away
	Spread	Nearest neighbour on average between 20-100m away
	Scattered/ split	Nearest neighbour on average more than 100m away

### Data collection

Data were collected over a period of approximately two years (27 March 2019 until 27 January 2021). An observation period of six weeks was allocated to each of the game reserves spanning over wet- and dry seasons (three weeks per season). Data collection consisted of behavioural observations as well as faecal samples. The faecal samples were collected to quantify fGCMs as a measure of adrenocortical activity.

The observer consistently approached the elephants in the same manner to conduct the behavioural observations. As soon as elephants were sighted, the observer positioned the vehicle appropriately (the observer did not approach the elephants too close (not closer than 30m)) for observations, while adhering to the reserve’s regulations. At the beginning of each observation session, the elephant being observed was identified, the sex and age was also recorded, as well as the history of the elephant. Age and sex were determined by either utilizing identification kits provided by the reserve or determined visually by size, height, and group association [64]. Furthermore, the distance between the elephants and the vehicle, as well as the grouping of the herd was also recorded (Table 3). Additionally, the occurrence of unusual behaviours towards conspecifics, heterospecifics and vehicles were recorded. During each observation session, 10-minute video recordings (focal samples) were taken of as many individual elephants visible as possible. These recordings were processed following the completion of the field work (see behavioural data processing).

#### Behavioural data processing

A total of 46 focal samples (10-minute focused observation periods) were processed for each of the eight game reserves (Table 6). Each focal sample consisted of the frequencies of detailed behaviours of only one elephant that was recorded continuously over the 10-minute period. Focal recordings were only processed when the elephants appeared to be undisturbed (the elephants were not responding to the vehicle) by the research vehicle. Focal samples from various individuals within a reserve were processed to ensure an accurate representation of each of the reserves. To account for seasonal effects, 23 focal samples were collected during both wet- and dry seasons, respectively.

An ethogram, compiled based on experience and literature (Table 4), was consulted to record detailed behavioural frequencies during each focal observation. Furthermore, the behaviours were selected and contextualized prior to the study to form various behavioural categories, which were thought relevant to studying the welfare of elephants. The behavioural categories were defined as Agonistic (interactions between elephants and included aggressive-, submissive- or threat behaviours); Arousal; Ambivalent; Assessing; Frustrated; Feeding, Drinking & Moving; Resting; and Social (affectionate behaviours between elephants such as rubbing, or touching behaviours). Most of these have been described by other authors [37, 41, 65]. The term Arousal [38] is used in this study to describe any behaviour that indicates the animal is responding to an external stimulus, and may escalate from mild alertness (e.g. head held high, ears spread) to threat or fear behaviour. Each of the behaviours were allocated to a specific behavioural category, of which some of the details could fit into more than one category. As a result of a relatively small sample size, five behavioural categories and only a few detailed behaviours (the behaviours that occurred more than 20 times in the data set) were used for this paper (Table 4). The detailed behaviours that fell into the frustrated behavioural category were not used for statistical analysis as some of the detailed behaviours only occurred in one or two reserves and will be investigated once more data have been collected.

**Table 4:** Description of the behavioural categories, into which the selected detailed behaviours were categorized.

Behavioural categories	Description	Selected detailed behaviour within the behavioural category	Description	Source
**Ambivalent**	Behaviours that seem inappropriate or irrelevant, often caused by a direct stimulus. These behaviours are displayed when an elephant is unsure of what action to take. Behaviours such as trunk twisting, tail raising, touching mouth, foot swinging, and trunk biting are associated with uncertainties.	Front foot swing	Lift the front foot slightly and swing back and forth.	[41, 65, 66]
		Biting own trunk	Trunk placed in own mouth and pulled down.	
		Trunk touch mouth	Elephant touches its mouth with its own trunk.	
		Trunk twist and twirl	Trunk folded onto itself, resulting in a twisted trunk that unwinds in a fast action.	
		Trunk in own mouth	Trunk is placed inside the mouth without pulling.	
		Hanging trunk rotate left and right	Trunk hangs straight while the tip is flicked to the left and right.	
**Ambivalent/ Body care**	A dual category was created to include behaviours that were difficult to contextualize. These behaviours include face touching/ brushing or swinging trunk to/between the legs.	Brushing face	Tip of trunk brushes over face. This is a fast action	Personal observations from zoo elephants
		Touching face	Touch any part of the face, including the ears with the tip of the trunk. Not a fast action.	
		Swing trunk to leg of foot	Trunk kept straight while being swung through front feet or touches one of the front feet.	
**Arousal**	**High arousal:** Behaviours induced by external stimuli, such as grouping together as a form of defence or fleeing. Loud vocalization, diarrhoea, and immediate temporal gland secretion can also occur during these situations.	Ears are spread	Both ears are spread out.	[37, 38]
	**Low arousal:** Behaviours occurring during unexpected or uncomfortable situations which triggers an alert state in elephants. The latter is characterized by a raised tail, head held high with ears spread out or retreat by walking warily with their ears out and tail up	Head held high	Head held high while the ears are spread out.	
**Assessing**	Gestures displayed by elephants to aid them in gaining sensory information about their surroundings. The latter includes smelling by lifting/holding the trunk in the direction of a stimulus or a sudden pause to gain auditory input. The elephant also uses its trunk and feet for physical investigation of the environment.	Smelling down	Trunk held in a relaxed position while the tip of the trunk is curled under and points in the direction of an object of interest.	[67–69]
		Lift trunk to smell	Lifts and holds the trunk up in an S-shape.	
		Sudden pause to listen	Sudden, short pause during any activity to listen.	
		Explore with trunk	Using trunk to explore an object of interest.	
**Frustrated**	When an elephant is confronted with unavoidable stress such as being blocked by a game viewing vehicle or other vehicles, or the absence of any healthy stimuli (lacking diverse vegetation types and water holes to stimulate movement of the elephants or not having enough elephants to interact with) causes it to display certain behaviours such as sweeping the ground with its trunk, head shaking, throwing item, trunk swirls, trunk on head or pushing objects with its head	Throwing item	Throw an object out of frustration, not related to feeding or play.	[53]
		Head shake	An abrupt shaking of the head.	
		Trunk swing	Trunk is flicked forward.	

### Faecal sampling

A total of 230 and 259 faecal samples were collected from females and males, respectively. The study aimed to collect samples that were representative of the entire population on a reserve (Table 6). Golf ball sized samples were collected from the centre of one or more boli, mixed and placed in plastic bottles less than two hours post-defecation. The samples were stored in a cooler box with ice bricks until it could be frozen on site at −18°C at the end of the day. The samples were kept frozen until further processing at the Endocrine Research Laboratory, University of Pretoria.

#### Faecal steroid extraction and fGCM assay

Faecal samples were lyophilized, pulverized and sifted using a nylon mesh strainer to remove fibrous material, as described by Fiess *et al 1999* [70]. Between 0.050–0.055 g of the faecal powder was then extracted with 80% ethanol in water (3 ml). The suspensions were vortexed for 15 min and subsequently centrifuged at 1500 *g* for 10 min. The supernatants formed were transferred into microcentrifuge tubes and stored at −20°C for further analysis.

The resulting extracts were measured for immunoreactive fGCM concentrations using an 11-oxoetiocholanolone enzyme immunoassay (EIA), detecting fGCMs with a 5*β*-3*α*-ol-11-one structure. This EIA has been shown to reliably detect adrenocortical function in African elephants [35]. Detailed assay characteristics, including full descriptions of the assay components and antibody cross-reactivities, have been provided by Möstl *et al 2002* [71]. Sensitivity (at 90% binding) of the assay was 1.5 ng/g dry weight (DW). Intra-assay coefficients of variation (CV) determined by repeated measurements of high and low value quality controls were 4.87% and 6.60%. Inter-assay CV determined by repeated measurements of high and low value quality controls were 14.76% and 15.73%. Serial dilutions of faecal extracts gave displacement curves that were parallel to the respective standard curve with a relative variation of the slope of the trend lines *<* 3%. All steroid concentrations are expressed per mass of faecal DW matter. All analyses were conducted at the Endocrine Research Laboratory, University of Pretoria with assay procedures following [72].

### Data analysis

Each of the eight reserves’ elephant populations have been described in Table 2. As a result of the small elephant population size on certain reserves (some reserves housing as few as eight elephants), occasionally more than one focal and faecal sample was processed of the same individual. It was not possible to always identify all individuals, especially in the larger groups and reserves such as reserve 2, and therefore data could well reflect behaviours of specific individuals.

Prior to the collection of the samples, targets for certain age and sex combinations (Table 5) were set. Some reserves only had a limited number of elephants that fall into the targeted age groups and sexes (in some reserves there was only one adult male and/or one adult female), which also led to multiple samples of the same individual in these categories (Table 5). We did not process focal samples of the same individual more than once in an observation session, to avoid pseudo-replication. Additionally, due to the vegetation and geography of some reserves, it was not possible to film and collect faecal samples from every individual in the herd (Table 6).

**Table 5:** Reserves with the number of study individuals that fall into the targeted age groups and sexes that could lead to multiple focal and faecal samples of the same elephant.

Reserve	Number of elephants	Adult		Sub-adult		Juvenile	
		Male	Female	Male	Female	Male	Female
1	25	3	5	5	5	7	0
2	3607*	452	Number of animals in breeding herds (also includes young bulls): 2992				
3	13	2	4	2	0	2	3
4	43	3	7	± 9	± 5	± 17	
5	12	1	2	1	4	2	2
6	29	3	5	9	6	4	2
7	9	1	2	1	1	2	2
8	8	3	1	0	2	2	0
**Targeted number of faecal samples**		20	20	6	6	6	6
**Targeted number of focal samples**		10	10	6	6	6	6

* Not all these elephants were observed.

**Table 6:** Total number of faecal and focal (10-minute video) samples collected for each reserve separated according to sex and age (adult; sub-adult; juvenile) of the individual.

Reserve	Sex	1	2	3	4	5	6	7	8
**Faecal samples**	**Male**	**37** (14;9;14)	**37** (18;13;6)	**25** (17;5;3)	**24** (13;7;4)	**35** (18;5;12)	**30** (15;14;1)	**32** (16;2;14)	**39** (32;0;7)
	**Female**	**26** (21;5;0)	**37** (24;10;3)	**23** (20;2;1)	**33** (24;6;3)	**42** (20;17;5)	**17** (9;5;3)	**23** (21;2;0)	**29** (10;19;0)
**Total**		**63**	**74**	**48**	**57**	**77**	**47**	**55**	**68**
**Focal samples**	**Male**	**29** (9;12;8)	**22** (10;10;0)	**27** (14;8;5)	**29** (6;14;9)	**20** (7;6;7)	**29** (13;13;3)	**27** (13;7;7)	**21** (15;0;6)
	**Female**	**17** (9; 8; 0)	**17** (9;5;3)	**19** (15;0;4)	**16** (8;8;0)	**26** (11;13;2)	**17** (8;7;2)	**19** (11;4;4)	**25** 12;13;0)
**Total**		**46**	39*	**46**	45**	**46**	**46**	**46**	**46**

*7 missing data

**1 missing data.

We used XLSTAT (version 2021.1) as well as R studio (R version 4.1.0) to perform the statistical analyses on the data collected for the study. Prior to any analysis, the Konglomorov-Smirnov test was used to test whether the behavioural (discrete) data complied with the assumption of normality. To test for normality of the faecal data (continuous), the Shapiro-Wilk test was performed. The behavioural and endocrine data both violated the assumption of normality, therefore the non-parametric Kruskal-Wallis test was utilised to test for significant differences between the eight reserves. Thereafter, Dunn’s Multiple comparison test was used as a post hoc and the Bonferroni correction was applied for multiple comparisons [73].

To provide a more in-depth understanding of the factors that could influence elephant behaviour and fGCM concentrations, generalized linear mixed models (GLMMs) were used to analyse the data (package: *lme4*). These factors will have been accounted for somewhat by including reserve as a random factor. It was attempted to include reserve as a fixed factor in the GLMMs but because each reserve has only one reserve size (and age structure, history, and GD/day), it was not possible to do so. By collecting additional data, the factors could be included in subsequent analysis.

The full model to analyse factors impacting the behaviour of elephants (behavioural categories and detailed behaviours) included elephant ID and reserve ID (to account for multiple samples of the same individual) as random factors, and reserve size, season, sex, age, social structure, elephant history and distance from observer as fixed effects. For the behavioural data, a negative binomial (*nbinom*) and Poisson distributions were specified as the ‘family’ for the distribution of the data. The full model to analyse factors impacting fGCM concentrations included elephant ID and reserve ID as random factors, and reserve size, season, social structure, elephant history, age, and sex as fixed effects. For the fGCM concentrations a Gamma distribution was selected to specify the ‘family’ for the distribution of the data.

The analysis for both behavioural and faecal data started with a full model, and then followed a backwards stepwise elimination procedure to define the final model for the response variables [74]. After each of the models were built, the “*overdisp fun”* function (package *lme4*) was used to check for overdispersion of the data. Where significant differences between the levels occurred, Tukey’s post-hoc test in the *multcomp* package was utilized. A 5% significance level was set as a guideline for determining significant effects.

### Ethical statement and permits

This project was approved by the Research Ethics Committee of the University of Pretoria permit. AEC Reference No.: REC254-19. Permission under Section 20 of the Animal Diseases Act, 1984 (act no 35 of 1984) to perform Research/Study was provided by the Department of Agriculture, Forestry and Fisheries of the Republic of South Africa. No authority permission was required for field access as all the research was conducted on private land and not government land.

## Results

### Behavioural data

**Fig 1:**
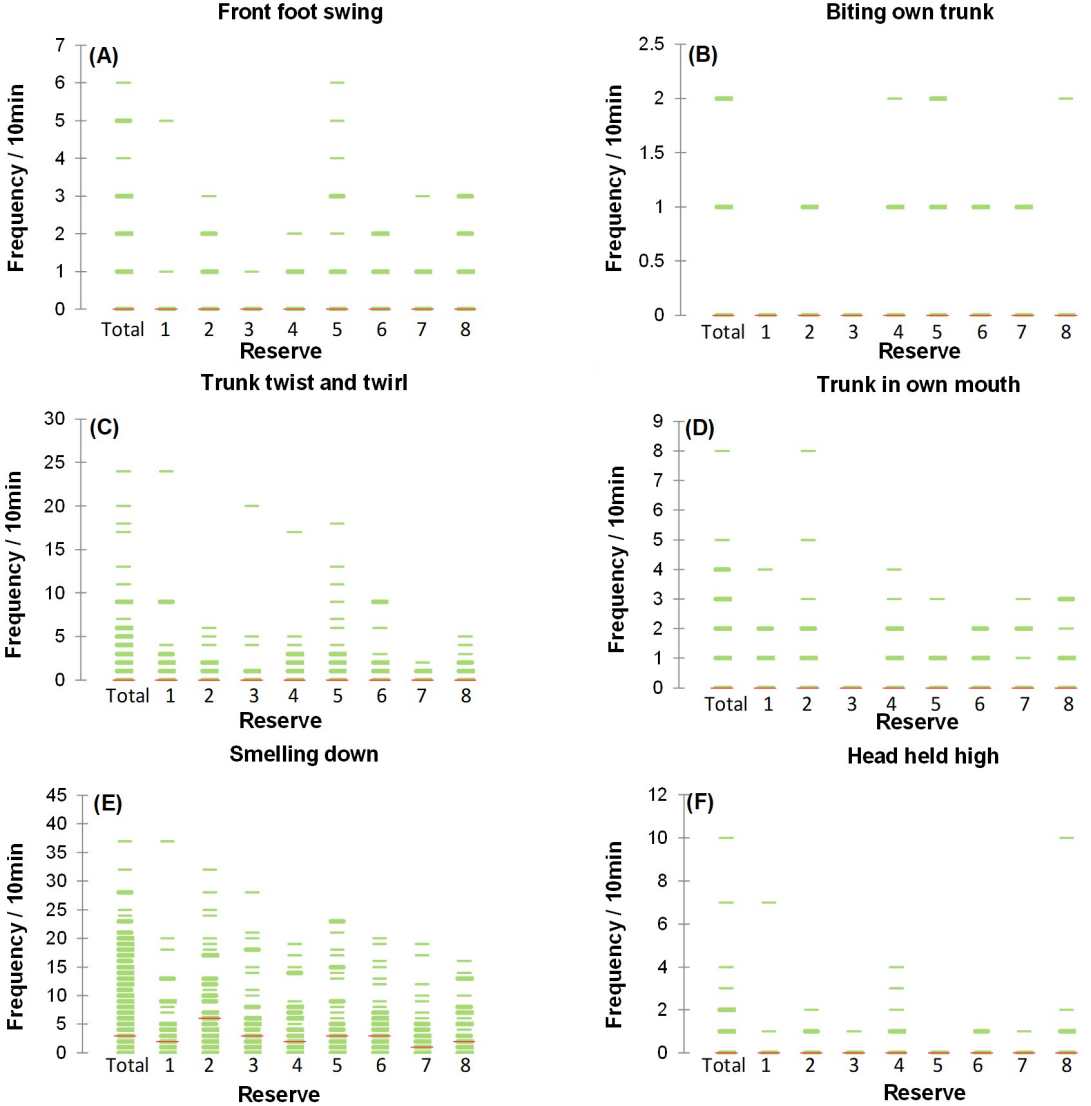
Strip plots that illustrate the influence of reserve on the frequency of the (A) Front foot swing, (B) Biting own trunk, (C) Trunk twist and twirl, (D) Trunk in own mouth, (E) Head held high and (F) Smelling down behaviours. The lines represent the distribution of frequencies observed for each focal sample and are plotted along the y-axis (for a given interval, the thicker or more tightly packed the strips, the more data there is).

There were significant reserve effects for Ambivalent (p = 0.003), Assessing (p = 0.001) and Arousal (p = 0.012) behaviours (Table 7). The elephants in reserve 5 showed significantly (p = 0.001) higher frequencies of Ambivalent behaviour than those in reserve 7 (Table 7). When investigating the detailed behaviours that fall within the Ambivalent behavioural category, we saw that elephants in reserve 8 had significantly (p = 0.001) higher frequencies of the “front foot swing” behaviour than elephants in reserve 3 (Fig 1A). As shown in Table 8, significantly higher frequencies were observed for the “biting own trunk” behaviour for elephants in reserve 5 in comparison to elephants in reserves 1 (p < 0.001), 3 (p < 0.001) and 8 (p = 0.001) (Table 8 and Fig 1B). elephants in reserve 5 also had significantly higher frequencies of the “trunk twist and twirl” behaviour than animals in reserve 7 (p < 0.001) (Fig 1C). Fig 1D shows significantly higher frequencies of the “trunk in own mouth” behaviour for animals in reserves 2 (p < 0.001) and 4 (p < 0.001) in comparison to the elephants in reserve 3 (Fig 1D). Furthermore, no significant differences were observed for both the “touch mouth with trunk” (p = 0.142) and “hanging trunk rotate left to right” (p = 0.228) behaviour when the elephants in the eight reserves were compared (Table 8).

**Table 7:** The influence of reserve on the frequencies of detailed behaviours (Median (frequency per 10 minutes), Range (maximum value is reported, minimum is 0 for all the data), and the p-value) grouped into five behavioural categories.

		Reserve							
		1	2	3	4	5	6	7	8
**Ambivalent behaviour**	Median	2 ^ab^	2 ^ab^	1 ^ab^	2 ^ab^	3 ^b^	1 ^ab^	1 ^a^	3 ^ab^
	Range	57	22	27	21	30	15	12	22
	p	**0.003**							
**Ambivalent/ Body care behaviour**	Median	2	2	1	2	2	1	1	1
	Range	27	15	16	12	35	50	38	43
	p	0.170							
**Assessing behaviour**	Median	2 ^a^	8 ^b^	4 ^ab^	3 ^ab^	5 ^ab^	4 ^ab^	2 ^a^	4 ^ab^
	Range	38	33	42	21	30	36	32	20
	p	**0.001**							
**Arousal behaviour**	Median	0 ^ab^	0 ^ab^	0 ^a^	0 ^ab^	0 ^ab^	0 ^ab^	0 ^ab^	0 ^b^
	Range	8	6	2	5	1	2	6	12
	p	**0.012**							
**Frustrated behaviour**	Median	0	0	0	0	0	0	0	0
	Range	2	2	2	2	2	3	2	5
	p	0.203							

n = 368 (total number of focal samples).

Medians with different letters within a row (behavioural category), differ significantly from one another (based on their mean ranks; a = reserve with the lowest mean rank).

**Table 8:** The influence of reserve on the frequencies of the detailed behaviours (Median (frequency per 10 minutes), Range (maximum value is reported, minimum is 0 for all the data) and the p-value.

		Reserve							
		1	2	3	4	5	6	7	8
**Front foot swing**	Median	0 ^ab^	0 ^ab^	0 ^a^	0 ^ab^	0 ^ab^	0 ^ab^	0 ^ab^	0 ^b^
	Range	5	3	1	2	6	2	3	3
	p	**0.003**							
**Biting own trunk**	Median	0 ^a^	0 ^ab^	0 ^a^	0 ^ab^	0 ^b^	0 ^ab^	0 ^ab^	0 ^a^
	Range	0	1	0	2	2	1	1	2
	p	**0.001**							
**Trunk twist and twirl**	Median	0 ^ab^	0 ^ab^	0 ^ab^	0 ^ab^	0 ^b^	0 ^ab^	0 ^a^	0 ^ab^
	Range	24	6	20	17	18	9	2	5
	p	**0.008**							
**Trunk in own mouth**	Median	0 ^ab^	0 ^b^	0 ^a^	0 ^b^	0 ^ab^	0 ^ab^	0 ^ab^	0 ^ab^
	Range	4	8	0	4	3	2	3	3
	p	**0.004**							
**Hanging trunk rotate left to right**	Median	0	0	0	0	0	0	0	0
	Range	25	2	0	4	17	4	0	2
	p	0.288							
**Trunk touch mouth**	Median	1	1	1	1	1	0	1	1
	Range	9	12	6	8	14	4	6	16
	p	0.142							
**Brushing face**	Median	1	0	0	1	0	0	0	0
	Range	16	11	7	5	24	4	8	15
	p	0.073							
**Touching face**	Median	0	0	0	0	1	0	0	0
	Range	10	12	3	6	11	6	7	15
	p	0.157							
**Swing trunk through legs or to foot**	Median	0	0	0	0	0	0	0	0
	Range	5	6	12	12	15	47	35	28
	p	**0.025**							
**Smelling down**	Median	2^bc^	6 ^c^	3^abc^	2^abc^	3^bc^	3^abc^	1 ^a^	2 ^ab^
	Range	37	32	28	19	23	20	19	16
	p	**0.000**							
**Sudden pause to listen**	Median	0	0	0	0	0	0	0	0
	Range	2	5	10	5	9	6	6	7
	p	0.314							
**Lift trunk to smell**	Median	0	0	0	0	0	0	0	0
	Range	4	5	11	8	7	3	7	7
	p	0.718							
**Explore with trunk**	Median	0	0	0	0	0	0	0	0
	Range	3	8	8	3	3	15	6	2
	p	0.051							
**Head held high**	Median	0 ^ab^	0 ^ab^	0 ^a^	0 ^ab^	0 ^a^	0 ^ab^	0 ^a^	0 ^b^
	Range	7	2	1	4	0	1	1	10
	p	**0.002**							
**Ears are spread**	Median	0	0	0	0	0	0	0	0
	Range	5	6	1	1	1	2	6	5
	p	0.270							

n = 368 (total number of focal samples).

Medians with different letters within a row (behavioural category), differ significantly from one another (based on their mean ranks; a = reserve with the lowest mean rank).

Elephants on reserve 2 showed significantly higher frequencies of Assessing behaviour in comparison to those in reserves 1 (p = 0.001) and 7 (p < 0.001) (Table 7). With regards to the detailed behaviour within the Assessing behavioural category, elephants in reserve 2 had significantly higher frequencies of the “smelling down” behaviour than those in reserves 7 (p < 0.001) and 8 (p = 0.001) (Fig 1E and Table 8). Furthermore, the elephants of reserve 5 revealed significantly (p = 0.002) higher frequencies of the “smelling down” behaviour than those on reserve 7 (Fig 1E and Table 8).

For Arousal behaviour, significantly (p < 0.001) higher frequencies were found for elephants in reserve 8 than those in reserve 3 (Table 7). Elephants in reserve 8 had significantly higher frequencies of the “head held high” behaviour than those in reserves 3 (p = 0.001), 5 (p < 0.001) and 7 (p = 0.001) (Fig 1F). No significant (p = 0.270) differences for the frequency of “ears are spread” behaviour were seen when the animals from the respective reserves were compared (Table 8).

No significant (p = 0.170) reserve effect was reported for the Ambivalent/ Body care behavioural category (Table 7). When the frequencies of “brushing face” (p = 0.073) and “touching face” (p = 0.157) behaviours were compared, no differences were observed between the elephants from the different reserves. A significant reserve effect was reported for the “swing trunk through legs or touch foot” (p = 0.025) behaviour, however after the Bonferroni correction was applied, no differences were found between the frequencies shown by the elephants on the eight reserves (Table 8).

Furthermore, the reserve did not have a significant (p = 0.203) effect on the frequency of Frustrated behaviour shown by the elephants and compared across the eight reserves (Table 7).

Age had a significant effect on various behavioural categories, as well as on the detailed behaviours. Sub-adults had significantly (p = 0.024) higher frequencies of Ambivalent/ Body care behaviour than adult elephants. In contrast, adults (p = 0.005) and sub-adults (p = 0.025) exhibited significantly higher frequencies of Assessing behaviour than juveniles. The adults showed significantly lower frequencies of Arousal behaviour than sub-adults (p = 0.007) and juveniles (p = 0.040). Furthermore, sub-adults exhibited significantly higher frequencies of the “front foot swing”(p = 0.029), “trunk in own mouth” (p = 0.041) and “swing trunk through legs or to foot” (p = 0.019) behaviours than adults. Juveniles demonstrated significantly higher frequencies of the “biting own trunk” (p = 0.027) behaviour than adults. Juveniles (p < 0.001) and sub-adults (p < 0.001) also had significantly higher frequencies of the “ears are spread” behaviour than adults. The adults (p = 0.006) and sub-adults (p < 0.001) exhibited significantly higher frequencies for the “smelling down” behaviour than juveniles.

Reserve size also had a significant effect on elephant behaviour, where elephants in the two large reserves displayed significantly higher frequencies of Arousal (p < 0.001) and Frustrated (p = 0.022) behaviours than those in the small reserves. Elephants on the two large reserves also showed significantly (p = 0.013) higher frequencies of the “trunk touch mouth” behaviour than those on the small reserves and significantly higher (p = 0.040) frequencies of the “ears are spread” behaviour than those roaming in medium sized reserves. Elephant history only affected the “front foot swing” behaviour, where ex-captive elephants showed significantly (p = 0.005) higher frequencies than wild elephants.

Sex of the elephants only influenced the “sudden pause to listen” behaviour, where females revealed significantly higher (p = 0.008) frequencies of this behaviour. Finally, season also affected elephant behaviour significantly, where elephants had significantly (p < 0.001) higher frequencies of Ambivalent/ Body care behaviours during the wet season. They also exibited higher frequencies of the “brushing face” (p = 0.002) and “swing trunk through legs or to foot” (p < 0.001) behaviours during the wet season. In contrast, elephants showed significantly (p = 0.036) higher frequencies of Arousal and “head held high” behaviours during the dry season.

### Faecal glucocorticoid metabolite (fGCM) concentration

**Fig 2:**
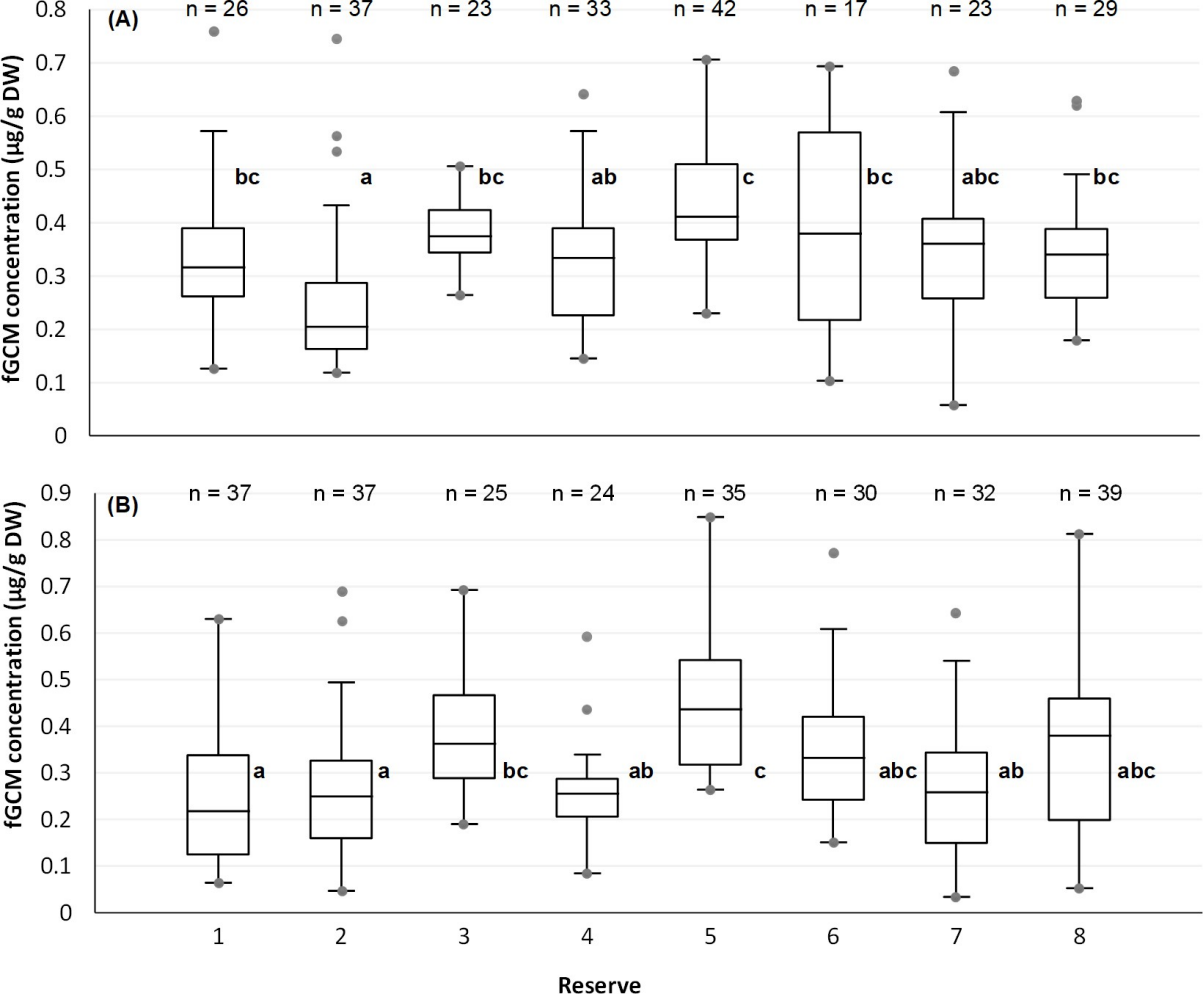
Influence of reserve on the faecal glucocorticoid metabolite (fGCM) concentration (μg/g DW) collected for (A) Female, and (B) Male elephants from eight reserves between March 2019 and January 2021. Different letters indicate significant differences between elephants of respective reserves (based on mean ranks, a = reserve with the lowest mean rank). The graph represents the minimum, 1st quartile, median, and 3rd quartile, together with both the limits (the ends of the "whiskers") beyond which values are considered anomalous. The limits were calculated as follows: Lower limit = Q1–1.5 (Q3—Q1); Upper limit = Q3 + 1.5 (Q3—Q1).

Previous studies reported differences in fGCM concentration between male and female elephants [59]; therefore, we investigated the effect of reserve separately for males and females. When examining reserve-related alterations in fGCM concentrations, female elephants in reserve 2 had significantly lower concentrations than animals in reserves 1 (p = 0.002), 3 (p < 0.001), 5 (p < 0.001), 6 (p < 0.001) and 8 (p = 0.001) (Fig 2A). The females in reserve 5 disclosed significantly higher fGCM concentrations than those roaming on reserves 2 (p < 0.001) and 4 (p = 0.001). The males from reserve 5 had significantly higher fGCM concentrations than those from reserves 1 (p < 0.001), 2 (p < 0.001), 4 (p < 0.001) and 7 (p < 0.001). Furthermore, bulls from reserve 1 had lower concentrations than those in reserves 3 (p = 0.001), and 5 (p < 0.001) (Fig 2B).

Furthermore, factors such as sex, reserve size, and season had a significant effect on the fGCM concentrations of the elephants. Female elephants presented significantly (p = 0.025) higher fGCM concentrations than males. Elephants in the medium sized reserves had significantly (p = 0.021) higher fGCM concentrations than those in the large reserves. Significantly (p = 0.040) higher fGCM concentrations were found for elephants during wet season in comparison to dry season. The history of the elephant as well as the social structure of the herd did not significantly affect the fGCM concentrations.

## Discussion

Defining welfare in a free-ranging population of elephants poses considerable challenges, as many factors vary across different reserves. This study is the first attempt at defining which behavioural categories could possibly be used to assess welfare and whether a similar trend is indicated by the fGCM concentration in free-ranging elephants, albeit in fenced, managed reserves. The results showed differences in some of the chosen behavioural categories, as well as in fGCM concentrations between the studied populations.

### Elephant behaviour

#### Reserve effect

Ambivalent behaiour (Table 7), often observed when an elephant is processing information or unsure of its next move in certain situations, was particularly high for the elephants in reserve 5, followed by those in reserve 8. Both these groups are composed of ex-captive elephants, except for the wild born calves. Other reserves with ex-captive elephants (reserves 3 and 6) did not display high frequencies of Ambivalent behaviour; however, reserve 6 only has one ex-captive elephant, and reserve 3 has a well-mixed population of ex-captive (31%) and wild elephants (69%). Reserve 5 had the highest frequencies of the “front foot swing”, “biting own trunk” and “trunk twist and twirl” behaviours, also known from zoo observations (Garaï pers. obs.), however, elephants in reserve 8 did not demonstrate significantly higher frequencies of these behaviours. Reserves 5 and 8 are medium and large reserves, however, they are comprised of an incomplete social structure. Both these reserves have a relatively low tourist density compared to the others, indicating that the history of the elephants might be a key factor playing a role in their behaviour, although other factors should not be ruled out.

Other factors may influence behaviour such as frequent darting events. For example, reserve 5 only houses one adult bull that spends most of his time with the herd, likely due to the application of the Gonadotropin Releasing Hormone (GnRH) vaccine and bonds formed during captivity. The adult bull is darted with the GnRH vaccine from a helicopter three times a year. It is possible this incursion could cause the higher frequencies of Ambivalent behaviours in the elephant population. Third highest rates of Ambivalent behaviour were observed in reserve 1, which keeps wild elephants. However, the social structure is incomplete, and some of the cows are immunocontracepted with Porcine zona Pellucida annually from a management vehicle.

The lowest frequencies of Ambivalent behaviour were found for the elephants in reserve 7. This reserve harbours a small, translocated elephant population with an incomplete social structure; therefore, one would expect them to be more nervous or apprehensive. However, the tourism density is the lowest of all reserves and the habitat allows them to take refuge from human disturbance in valleys and hills. In addition, the elephants were slowly habituated to vehicles, which keep a larger distance from the elephants than the vehicles on other reserves. An adult bull was introduced to this family unit shortly after their translocation. It is speculated that this might have eased the habituation process of the herd, as the bull was already confident around vehicles. These results suggest that management and tourism viewing protocols seem to play an important role in influencing elephant behaviour.

Many traditional elephant behavioural studies have focused on key, well understood behaviours to investigate welfare of elephants. If we only looked at these traditionally defined Ambivalent behaviours [14, 75] we could have missed important signals. For example, the elephants of reserve 3 displayed low or no frequencies of the “front foot swing”, “biting own trunk”, and “trunk in own mouth” behaviours, however they did show the “trunk twist and twirl” behaviour often.

Assessing behaviour was seen significantly more in reserve 2 (reference reserve), especially the detailed behaviour of “smelling down”. Reserve 2 is adjacent to KNP with access to over 100 000 ha of land, however, it has a high tourist density (game drives and self-drives), and hunting is permitted. In addition, this reserve has one main tar road that runs right down the centre of the reserve with many delivery vehicles which are known to occasionally speed past the elephants. It will be important to investigate individual variation in behaviour relative to the distance from this road in future, but it is apparent it cannot be deemed an undisturbed population. Relatively high tourist density and the way the elephants are approached (some tourists lack knowledge of elephant behaviour) as well as the hunting practice may cause elephants to assess their environment more.

Arousal behaviour and the detailed behaviour of “head held high”, indicating they were alert to disturbance, was higher for the elephants in reserve 8, comprised of ex-captive elephants. Interestingly the other reserves with ex-captive elephants, reserves 3 and 5, did not show this behaviour. Reserve 8 has a vastly different habitat than the one the elephants were previously in, and the generally very low vehicle density may have made the elephants more alert when one was nearby, despite them having been once trained and used to people. This would indicate that ex-captive elephants can be dishabituated to people, allowing reintegration back into the wild.

For both Ambivalent/Body care behaviour and Frustrated behaviour, no significance differences were reported among any of the reserves. The elephants in reserves 5 and 8 (predominantly ex-captive) had the highest frequencies of Ambivalent/ Body care behaviour. The detailed behaviours “brushing face“, “touching face” and “swing trunk through legs or to foot” are typical behaviours of zoo elephants implying nervousness or unease (Garaï & Mitchell as per video obs.), and interestingly, the elephants of reserve 8 demonstrated the highest, though not significantly different frequencies of these behaviours; whether this indicates that they are still apprehensive, or whether these are learned behaviours from their captive days, cannot be assessed. It would be interesting to see if these behaviour patterns are passed on to the next generation, perhaps as part of the group culture [76] or epigenetic make-up [77]. As a result of the fenced reserves, elephants may only have the opportunity to learn a limited range of behaviours, which can result in apparently different ‘cultures’ [78].

It is evident that not all the elephant populations on the various reserves have the same behaviours. Some display high frequencies of a couple of behaviours, and some behaviours are even absent in certain elephant populations. It is now widely accepted that individual animals of a population have personalities [79], that they react differently to stressors, which have varying effects on cognition and memory [80], and that individuals develop varying coping styles for stress [81]. Therefore, one would expect individual behaviour expression. However, even if one individual experiences compromised welfare in a group, this could have implications on the other elephants as personality and learned behavioural reaction can be passed on to the next generation and affect the other elephants in the group [82]. An interesting example was seen on reserve 1, where a behaviour not previously described by other authors, “hanging trunk rotate left and right”, and which has been observed in zoo elephants frequently (Garaï & Mitchell as per video obs). This behaviour was performed by one female, whose history is unknown. Therefore, it is important to consider all detailed behaviours of any selected category when assessing the welfare status to account for individual and even population variation.

Rather unexpectedly, the elephants of the reference reserve (reserve 2) did not differ much in their behaviour from the those on other reserves. These elephants expressed relatively frequent Assessing and Ambivalent behaviour. Unfortunately, there is no undisturbed elephant population in South Africa to act as a true reference population, as is probably the case in most high tourism range states, and tourism has been revealed to be reflected in higher fGCM concentrations in elephants [83]. This highlights the fact that all elephants will potentially experience some form of disturbance due to human population growth and impact on elephant habitat, and increasing Human-Elephant-Conflict, and this may change their behaviour patterns.

#### Other factors influencing elephant behaviour

Sub-adults had significantly higher frequencies than adults for Arousal and Ambivalent/ Body care behaviours, expressed by several of the detailed behaviours. Even juveniles displayed more Arousal behaviours than adults, particularly “biting own trunk” and “ears are spread”. Juveniles would not have the experience to assess danger yet, so one would expect their Ambivalent behaviour to be low, whereas sub-adults do perceive danger, but have possibly not yet developed a coping strategy [84] as well as adults have, or they do not have sufficient experience to analyse the danger and would, therefore, express unease. In contrast, adult elephants with the added responsibility of looking after the young [85] would try and assess a situation as soon as possible for danger, as reflected in the significantly higher Assessing behaviour including “smelling down”. Females appeared to perform more “sudden pause and listen” than did males. This supports the notion that females have a higher responsibility in protecting their family than do males, and therefore would try and assess a situation as soon as possible to avoid danger [86].

The elephants in the two large reserves (2 and 8) appeared to have significantly higher frequencies of Arousal and Frustration behaviours than elephants in the small reserves, although one would expect a large reserve to provide a more calming effect. These results could be due to the other variables, such as the captive history of the elephants in reserve 8, or the high tourism volume and hunting permitted on reserve 2. To analyse this further would require more data and is beyond the scope of this study. Although ex-captive elephants on reserve 8 had higher frequencies for some of the behaviours as discussed above, overall ex-captive versus wild elephants only indicated an effect for one detailed behaviour (“front foot swing”), but again more data is required to tease out the variables.

Season appeared to influence Ambivalent/ Body care, with the two detailed behaviours, “brushing face” and “swing trunk through legs or to foot”, indicating significantly higher frequencies during the wet season, which in six out of the eight reserves coincided with the reserves’ high tourism season. The thicker vegetation during the wet season does not allow the elephants the same amount of visibility and ability to hear when potential stressors approach which could also have caused the higher frequencies of these behaviours. The higher abundance of insects during the wet season could also have contributed to the higher Ambivalence/Body care frequencies due to increased annoyance experienced by the elephants. However, as both detailed behaviours have been documented for zoo elephants, the results may be due to a few individuals displaying these behaviours.

### Faecal glucocorticoid metabolite (fGCM) concentration

#### Reserve effect

The fGCM levels were the highest for elephants in reserve 5. This supports the behavioural results, where these ex-captive elephants showed high frequencies of Ambivalent and Ambivalent/ Body care behaviour. Similar findings were published on African wild dogs (*Lycaon pictus*) where respective levels of glucocorticoids or their metabolites were also higher in captive animals [87]. However, it should be mentioned that although fGCM concentrations are comparatively higher in some of the ex- captive elephants, hormone metabolite values are still within the comparable range of previously determined baseline values for male and female African elephants [14, 83, 88].

Furthermore, the elephant cows from reserve 2 had significantly lower levels of fGCM than those in five other reserves and the bulls had lower levels of fGCM than the animals in two other reserves. It is interesting to note that the elephants on reserve 2 exhibited high frequencies of some of the behaviours. For example, they had high frequencies of Ambivalent behaviour, “trunk in own mouth” as well as the “touching face” and “brushing face” behaviours, however, these behaviours were not related to elevated fGCM concentrations. Their higher behavioural frequencies may have been caused by factors such as the high tourist density, which include game drives and self-drives and possibly the type of social relationships they have with other elephants on the reserve. This also emphasizes the importance of including both behavioural data as well as quantifying of stress-related physiological biomarkers like glucocorticoids to best evaluate elephant welfare.

#### Other factors influencing the fGCM concentrations of elephants

Reserve size also appeared to affect fGCM concentrations in the study animals, with medium sized reserves presenting significantly higher fGCM concentrations compared to the small and large sized reserves in our study. However, both the medium sized reserves have elephants with a captive history and incomplete social structure, which could explain the higher concentrations.

The data on fGCM concentrations revealed overall sex-related differences, with higher levels for females than for males, which is in line with other studies [83, 89]. These differences may be attributed to females having more responsibilities in a population than males [90], especially the adult females with offspring. Sex-related differences could also be attributed to differences in physiology and diet [27, 59].

Season also influenced the fGCM concentrations, with higher levels occurring in the wet season. This is somewhat unexpected as previous studies showed significantly higher fGCM concentrations in the dry season than in the wet season [36, 83]. A possible explanation for the seasonal related differences in fGCM concentrations could be attributed to the small population sizes on the reserves as well as artificial waterholes. Naturally, water and vegetation availability would become limited during the dry season. However, in reserves that control their elephant population size (less pressure on vegetation) and water availability, elephants may not experience the dry season as challenging, as they would in bigger, more natural systems [34, 91]. Even though our data did not allow us to test for the impact of tourism pressure on the fGCM levels of elephants, it could also have played a role. During the data collection periods, six out of the eight reserves’ wet season coincided with the reserves’ high tourism season. Szott *et al* 2020 [83] reported significantly higher fGCM concentrations in the individually identified elephants during the high monthly tourism levels in Madikwe Game Reserve. As mentioned previously, the thick vegetation during the wet season does not allow for the same level of visibility and auditory signals for approaching stressors and could have caused the higher levels. It is also important to note that even though the model revealed a significant difference between the seasons, those differences might not be biologically relevant as the medians were virtually the same.

Although there were no significant differences in fGCM levels between incomplete and complete social groups across all reserves, this does not rule out the possibility that incomplete groups may have compromised welfare, as alterations in glucocorticoid concentrations are not the sole indication of welfare quality. Other factors such as tourist numbers and management interventions can have an impact on fGCM concentrations [83] and thus should be considered in subsequent studies.

## Conclusions

This first step in studying welfare of elephants on fenced reserves was to find possible behavioural parameters to assess welfare, and this has been achieved. The five behavioural categories, Ambivalent, Ambivalent/ Body care, Arousal, Assessing and Frustrated behaviours can be utilised. However, it is very important to consider all detailed behaviours within the respective categories, as these differ between reserves and possibly individuals. The main variables significantly influencing behaviour and fGCM levels that were analysed were reserve, season, sex, history of the elephant and size of reserve.

The results indicated possible variables that should be studied more in depth, such as tourism density, specifically game drive protocols and distance of vehicles to the elephants, as well as management interventions (e.g. frequent darting). Previously collected unpublished data (Elephant Specialist Advisory Group—ESAG) suggest that incomplete social groups may also display different behaviour to socially complete groups, and this study seems to support that hypothesis.

The authors are aware that the amount of data obtained to decipher the causes possibly influencing welfare are limited, and this study indicates only trends, allowing for further hypotheses to be tested, such as history, social completeness, or tourism density, but also management approach and interventions. Some of these possible variables are the subject of the second part of the welfare project, which is continuing.

## Supporting information

S1 File (DOCX)

S1 Data (XLSX)
