# Supplementary material for: Developing welfare parameters for African elephants (Loxodonta africana) in fenced reserves in South Africa
Source: PLoS One. 2022 Mar 24;17(3):e0264931. doi: 10.1371/journal.pone.0264931 (PMC8947097; doi:10.1371/journal.pone.0264931)
Supplement: S1 File — (DOCX) [file pone.0264931.s001.docx]

**Ethical statement**

**Ethics Approval**

Approved by the University of Pretoria, Faculty of Veterinary Science, Animal Ethics Committee.

AEC Reference No.:	REC254-19

Title:	New title: Using Cortisol & Reproduction Hormone levels in Dung to Develop Welfare Parameters to Guide Management on Free Ranging Elephants and the Reintegration of Captive Elephants back to the Wild. Old title: Developing Welfare Parameters for Elephants on Reserves in South Africa

Researcher:	Dr ME Garai

Dear Dr ME Garai,

The Amendment as supported by documents received between 2020-04-16 and 2020-05-04 for your research, was approved by the Animal Ethics Committee on its quorate meeting of 2020-05-04. This project was approved by the Research Ethics Committee of the University of Pretoria permit REC254-19. (Permit uploaded)

**Faecal Sample Collection Approval**

Approval by the Department of Agriculture, Forestry and Fisheries of the Republic of South Africa.

Permission under Section 20 of the Animal Diseases Act, 1984 (act no 35 of 1984) to perform Research/Study was provided by the Department of Agriculture, Forestry and Fisheries of the Republic of South Africa.

Dear Dr Garai

CONDITIONS FOR RESEARCH IN TERMS OF SECTION 20 OF THE ANIMAL DIESEASES ACT, 1984 (ACT NO 35 OF 1984)

Titles of research project / study: Using Cortisol & Reproduction Hormone levels in Dung to Develop Welfare Parameters to Guide Management on Free Ranging Elephants and the Reintegration of Captive Elephants back to the Wild.

Your application, requesting permission under Section 20 of the Animal Diseases Act, 1984 (Act no 35 of 1984) to perform the research project or study stipulated above, refers.

1. Based on the information in your application, the Director of Animal Health has no objection to this study. (Permit uploaded)

**Field Site Access**

No authority permission was required for field access as all the research was conducted on private land and not government land.

**Declaration of interests**

The authors declare no conflict of interests.
